# Supplementary material for: Evaluation of the immune status of dogs vaccinated against rabies by an enzyme-linked immunosorbent assay using crude preparations of insect cells infected with a recombinant baculovirus encoding the rabies virus glycoprotein gene
Source: PLoS One. 2024 Dec 3;19(12):e0314516. doi: 10.1371/journal.pone.0314516 (PMC11614288; doi:10.1371/journal.pone.0314516)
Supplement: S1 Table — (DOCX) [file pone.0314516.s001.docx]

**Supplementary Table 1. Details of the samples with RFFIT and ELISA values**

| **Sample**  **ID** | **Age** | **Gender** | **Breed** | **Vaccine**  **brand** | **Time period** | **RFFIT**  **Titre (IU/mL)** | **ELISA**  **Corr OD** | **ELISA**  **PP values** |
| --- | --- | --- | --- | --- | --- | --- | --- | --- |
| **1** | 4y 7 m | M | Labrador Retriever | V1 | 40 d | 1 | 0.788 | 63.8 |
| **2** | 5y 1m | M | ND | V1 | 4m 25d | 16 | 1.25 | 82.25 |
| **4** | 4y | M | Bull Mastiff | V4 | 3m 10d | 4 | 0.6525 | 51.15 |
| **5** | 6m | M | Beagle | V2 | 2m 5d | 2 | 0.571 | 36.54 |
| **6** | 6y | M | Labrador Retriever | V5 | 5m 24 d | 8 | 0.7085 | 48.64 |
| **7** | 11y | F | Dachshund | UK | 3m 5d | 2 | 0.689 | 67.41 |
| **8** | 1y 8m | M | Golden Retriever | V4 | 1m 13d | 2 | 0.849 | 68 |
| **9** | 11m | M | Siberian Husky | V4 | 1m 13d | 1 | 0.5365 | 43 |
| **10** | 7m | M | Labrador Retriever | V2 | 3m 12d | 1 | 0.412 | 28.28 |
| **11** | 5y | F | Pug | UK | 7m 19d | <0.5 | 0.297 | 28.11 |
| **12** | 5y | M | Pug | UK | 3m 11d | <0.5 | 0.211 | 25.11 |
| **13** | 1y 7m | M | Pug | UK | 4m 14d | <0.5 | 0.419 | 36.11 |
| **14** | 3y 8m | M | Boxer | V5 | 1m 25d | 1 | 0.472 | 38.26 |
| **15** | 3y 6m | F | Boxer | V5 | 1m 25d | 1 | 0.5275 | 42.76 |
| **16** | 3y 6m | F | Boxer | V5 | 1m 25d | 2 | 0.7 | 60.71 |
| **17** | 5y 6 m | M | English Cocker Spaniel | V2 | 6m 4d | 1 | 0.8685 | 53.24 |
| **18** | 7y 7m | M | Labrador Retriever | V2 | 9 days | 2 | 0.5505 | 45.25 |
| **20** | 1yr | M | Labrador Retriever | V2 | 3m 11d | 2 | 0.595 | 53.14 |
| **21** | 14 y | F | Maltese Terrier | V1 | 23d | 2 | 0.54 | 18.59 |
| **23** | 10y | F | Beagle | V7 | 3m 28d | 4 | 0.692 | 77.03 |
| **24** | 11y | M (N) | Beagle | V7 | 3m 28d | 0.5 | 0.703 | 68.78 |
| **25** | 4y | F(N) | ND | V7 | 3m 28d | 2 | 0.576 | 47 |
| **26** | 3y 11m | M | Cocker Spaniel | V2 | 10 m 23d | 0.5 | 0.452 | 36.64 |
| **27** | 1y 4m | F | ND | V2 | 10 m 23d | 2 | 0.9165 | 62.92 |
| **28** | 4y 5m | F(N) | Labrador Retriever | V6 | 1m 6d | 2 | 0.659 | 45.24 |
| **32** | 2y 4m | F | Labrador Retriever | V4 | 2m 4d | 4 | 0.499 | 34.24 |
| **33** | 2y 11 m | F | German Shepherd | UK | 1m 22d | 2 | 0.6425 | 44.11 |
| **34** | 11y | F | Coton de Tullear | V3 | 8d | 2 | 0.6115 | 41.98 |
| **35** | 10y | M | Coton de Tullear | V3 | 4m 23 d | 2 | 0.5615 | 45.52 |
| **36** | 7y 2m | M | Labrador Retriever | V2 | 5m 21d | 2 | 0.5395 | 43.73 |
| **38** | 6y | M | Labrador Retriever | V6 | 2m 24d | 1 | 0.761 | 55.24 |
| **39** | 5y | M | Pomeranian | V3 | 6m 7d | 2 | 0.6895 | 55.89 |
| **40** | 2y 2m | M | Labrador Retriever | V2 | 1m 18d | 1 | 0.6135 | 49.73 |
| **42** | 3y 10m | F(N) | ND | V2 | 10m 4d | 1 | 0.893 | 72.39 |
| **43** | 7y | F | Pomeranian | V2 | 5m 15d | 0.5 | 0.774 | 75.73 |
| **44** | 1y | M | Siberian Husky | V2 | 4m 3d | <0.5 | 0.2245 | 21.24 |
| **45** | 8y | M | English Bulldog | V6 | 5m 3d | 0.5 | 0.6225 | 50.46 |
| **46** | 9y 7m | M | Pomeranian | V1 | 3m 27d | <0.5 | 0.3515 | 33.42 |
| **48** | 8y | M(N) | German Shepherd | V4 | 2d 30d | 0.5 | 0.7705 | 62.46 |
| **49** | 4y 6m | F(N) | German Shepherd | V2 | 2m 30d | 0.5 | 0.5305 | 43 |
| **50** | 4y | M(N) | Golden Retriever | V6 | 6m 1 d | 1 | 0.649 | 52.61 |
| **51** | 4y 8 m | M | Labrador Retriever | V4 | 1m 12d | <0.5 | 0.2295 | 21.72 |
| **52** | 8m | M | Beagle | V2 | 3m | 4 | 0.749 | 45.69 |
| **54** | 2y | F(N) | Irish Terrier | V2 | 9m 2d | 1 | 1.821 | 62.29 |
| **55** | 7y | M(N) | Pomeranian | V6 | 8m 12d | 0.5 | 0.4335 | 30.3 |
| **56** | 8y | F | Spitz | V7 | 2m 10d | 4 | 0.6865 | 48.86 |
| **59** | 3y 2 m | M(N) | Pug | V2 | 3m 14d | 1 | 0.8685 | 60.71 |
| **61** | 6y 1m | F | Cocker Spaniel | V7 | 9m 2d | 2 | 0.7755 | 53.24 |
| **62** | 5y 1m | M | Labrador Retriever | V3 | 1m 1d | 0.5 | 0.49 | 45.25 |
| **63** | 6y 4m | M | German Shepherd | V1 | 1m 4d | 0.5 | 0.6355 | 53.14 |
| **64** | 6m | F | Labrador Retriever | UK | 1m 26d | <0.5 | 0.1965 | 18.59 |
| **65** | 2y 11m | F(N) | German Shepherd | V3 | 1m 22d | 2 | 0.5385 | 42.21 |
| **66** | 11y | F | Coton de Tullear | V3 | 8d | 2 | 0.693 | 54.33 |
| **67** | 10y | M | Coton de Tullear | V2 | 4m 23 d | 2 | 0.455 | 31.27 |
| **68** | 7y 2m | M | Labrador Retriever | V3 | 5m 21d | 2 | 0.5995 | 41.16 |
| **69** | 3 y 11m | M | Boxer | V6 | 158d | 1 | 0.9425 | 57.5 |
| **70** | 6y | M(N) | Labrador Retriever | V3 | 2m 24d | 1 | 0.56 | 43.9 |
| **71** | 5y | M | Pomeranian | V2 | 6m 7d | 2 | 0.44 | 26.84 |
| **73** | 3y 9m | M | Labrador Retriever | V2 | 10m 4d | 0.5 | 0.596 | 40.92 |
| **75** | 7y | F | Pomeranian | V6 | 5m 15d | 0.5 | 1.0795 | 84.63 |
| **77** | 8y | M | English Bulldog | V1 | 5m 3d | 0.5 | 0.749 | 58.72 |
| **78** | 9y 7m | M | Pomeranian | V1 | 3m 27d | <0.5 | 0.3865 | 39.65 |
| **79** | 9y 9m | M | Pomeranian | V4 | 153 d | 0.5 | 1.1755 | 71.72 |
| **80** | 8y | M(N) | German Shepherd | V7 | 2d 30d | 0.5 | 0.445 | 39.88 |
| **81** | 5y | F | Labrador Retriever | V6 | 28d | 0.5 | 1.03 | 70.71 |
| **83** | 4m | M | Labrador Retriever | V3 | 1m 24d | <0.5 | 0.117 | 11.07 |
| **84** | 1y 6m | F | Beagle | V5 | 6m 2d | <0.5 | 0.3515 | 33.42 |
| **86** | 2y | M | Cocker Spaniel | V3 | 01m | 1 | 0.6315 | 43.35 |
| **87** | 4y 8m | F | Labrador Retriever | V3 | 02m 11d | 2 | 0.4655 | 31.96 |
| **88** | 2y 2m | M | Labrador Retriever | V2 | 02m08d | 4 | 0.866 | 59.45 |
| **89** | 3y 8m | M | Cocker Spaniel | V1 | 5m | <0.5 | 0.4465 | 35.05 |
| **90** | 1y 10m | M(N) | Labrador Retriever | V7 | 1m 12d | <0.5 | 0.443 | 42.57 |
| **91** | 10y | M(N) | Cocker Spaniel | UK | 4m 3d | 0.5 | 0.6135 | 42.12 |
| **92** | 3y | M | Pug | V3 | 4m 3d | 4 | 0.6385 | 62.85 |
| **93** | 5y 8m | M | Golden Retriever | V6 | 3m 23d | 2 | 0.6925 | 47.54 |
| **95** | 5m | M | Labrador Retriever | V1 | 30d | 1 | 0.4315 | 26.32 |
| **96** | 14y 6m | F | German Shepherd | V1 | 06m 6d | <0.5 | 0.5185 | 40 |
| **97** | 1y 11m | M(N) | Labrador Retriever | V5 | 2m 3 d | <0.5 | 0.495 | 60.76 |
| **98** | 1y 6m | M | Pug | V4 | 2m 2d | 1 | 0.488 | 51.64 |
| **99** | 1y 6m | F | Beagle | V4 | 1m 1d | 0.5 | 0.7475 | 58.6 |
| **101** | 1y | F(N) | Russian Bolanka | V2 | 8m 12d | <0.5 | 0.306 | 28.96 |
| **104** | 5y | F | Pug | V1 | 2m 9d | 0.5 | 0.615 | 60.17 |
| **105** | 1y 11m | M(N) | Labrador Retriever | V1 | 2m 20d | 0.5 | 0.626 | 49.07 |
| **106** | 14y 6m | F | Maltese Terrier | V3 | 6m 7d | 0.5 | 0.5205 | 40.8 |
| **107** | 13y | M | Poodle | V2 | 1m 9d | 0.5 | 0.525 | 41.27 |
| **108** | 1y 10m | M | ND | V3 | 1m 10d | 1 | 0.65 | 44.62 |
| **109** | 1y 10m | F | Pomeranian | V2 | 6m 25d | 4 | 0.55 | 33.55 |
| **110** | 3y 4m | F | Golden Retriever | V2 | 1m 2d | 2 | 0.8605 | 52.5 |
| **111** | 2y 3m | M | Labrador Retriever | V1 | 32d | 0.5 | 1.4825 | 90.45 |
| **112** | 2y 6 m | F | Schi Tzu | V1 | 3m 15d | 2 | 0.5895 | 40.42 |
| **113** | 3y 9m | M | Cocker Spaniel | V4 | 1m 8 d | 4 | 0.65 | 61.32 |
| **114** | 12y | M(N) | ND | V3 | 1m 4d | 1 | 0.9995 | 78.36 |
| **115** | 10y | F(N) | ND | V1 | 1m 4d | 2 | 1.1345 | 88.94 |
| **116** | 1y 1m | M | Siberian Husky | V5 | 1m 27d | <0.5 | 0.274 | 25.93 |
| **117** | 1y | F(N) | Russian Bolanka | V3 | 21d | 4 | 0.641 | 44.96 |
| **118** | 9m | M | Pomeranian | V1 | 3m 24d | <0.5 | 0.2025 | 19.16 |
| **119** | 9m | M | Labrador Retriever | V3 | 02m 6d | 1 | 0.5435 | 37.31 |
| **120** | 8y 2m | F | Labrador Retriever | V3 | 1m 3d | 1 | 0.8485 | 60.39 |
| **121** | 1y 11m | M | Dachshund | V6 | 1m 3d | 1 | 0.7505 | 51.52 |
| **123** | 4y 10m | M | Maremma | V6 | 5m 2d | 2 | 0.896 | 65.49 |
| **124** | 1y 8m | M | ND | V6 | 5m 02d | <0.5 | 1.021 | 53.73 |
| **125** | 1y 8m | F | ND | V2 | 5m 2d | <0.5 | 0.326 | 35.8 |
| **126** | 4y 5m | F | Golden Retriever | V3 | 30d | 0.5 | 0.3 | 21.04 |
| **127** | 1y 11m | M | Labrador Retriever | V3 | 8m 13d | <0.5 | 0.2785 | 25 |
| **128** | 9y 9 m | F | Labrador Retriever | UK | 1m 6d | 1 | 0.754 | 53.66 |
| **129** | 9m | M | Pomeranian | V3 | 4m 8d | <0.5 | 0.2445 | 23.14 |
| **130** | 5y | M | ND | V1 | 1m7d | 4 | 0.735 | 65.49 |
| **132** | 4y 6m | M | Chihuahua | V7 | 2m 2d | 1 | 0.753 | 67.31 |
| **135** | 2y 6m | M | Dalmatian | V2 | 6m 12 d | <0.5 | 0.3395 | 20.71 |
| **136** | 3y 10m | M(N) | Beagle | UK | 1m 3d | 8 | 0.733 | 51.42 |
| **138** | 5y 2m | M | Pug | V4 | 23d | 0.5 | 0.69 | 48.4 |
| **139** | 5y | M(N) | German Shepherd | V4 | 5m 4d | 1 | 0.5825 | 39.39 |
| **140** | 2y | F(N) | Siberian Husky | V3 | 5m 5d | 1 | 0.572 | 40.12 |
| **141** | 5y 6m | F | Beagle | V2 | 3m 15d | 0.5 | 0.689 | 46.6 |
| **142** | 3y | M | Labrador Retriever | V3 | 9m 20d | <0.5 | 0.369 | 25.79 |
| **143** | 6m | M | English Bulldog | V6 | 11m 3d | 1 | 0.5 | 42.6 |
| **145** | 3y | M | Pug | V2 | 1m25d | <0.5 | 0.364 | 36.12 |
| **146** | 3yr 10m | M(N) | Labrador Retriever | V2 | 6m 5d | 8 | 0.695 | 65.83 |
| **148** | 7y 6m | M | Dalmatian | V7 | 12m 1d | 1 | 0.7695 | 53.79 |
| **149** | 4yr 11m | M | Beagle | V2 | 20d | <0.5 | 0.2925 | 24.67 |
| **151** | 6y | M | Labrador Retriever | V2 | 6m 12d | <0.5 | 0.475 | 33.24 |
| **152** | 9y | M | Labrador Retriever | V6 | 6m 12d | 0.5 | 0.537 | 37.57 |
| **153** | 1y 8m | M | ND | V6 | 29d | 2 | 0.224 | 15.71 |
| **154** | 1y 8m | F | ND | V3 | 29d | <0.5 | 0.733 | 51.6 |
| **155** | 1y 1m | F | Labrador Retriever | V1 | 20d | <0.5 | 0.2865 | 25.18 |
| **156** | 4y 8m | M(N) | Labrador Retriever | V6 | 27d | 1 | 0.781 | 54.59 |
| **157** | 6y | M | Labrador Retriever | V8 | 6m 15d | 0.5 | 0.5955 | 40.88 |
| **158** | 2y 5m | F(N) | Golden Retriever | V2 | 18d | 0.5 | 1.0965 | 76.92 |
| **160** | 8y | M(N) | Labrador Retriever | V2 | 27d | 4 | 1.109 | 67.66 |
| **161** | 8y | F(N) | Labrador Retriever | UK | 27 d | 8 | 1.263 | 77.05 |
| **162** | 8yr | F | Rottweiler | V2 | 1m 16d | 4 | 0.581 | 54.99 |
| **163** | 4y 9m | F | Havanase | V5 | 30d | 0.5 | 0.5685 | 39.74 |
| **164** | 5y 6m | F | Labrador Retriever | V6 | 31d | 2 | 0.466 | 63.61 |
| **165** | 1y 8m | F | ND | V4 | 1m 6 d | 4 | 0.882 | 53.81 |
| **166** | 10y | F | American Silk Terrier | V5 | 06m 28d | 1 | 0.6715 | 46.94 |
| **167** | 1y 9 m | M | Pug | V2 | 5m 10d | 2 | 0.724 | 50.61 |
| **168** | 6y | M(N) | Schnoodle | V1 | 1m 26d | 2 | 0.621 | 43.56 |
| **169** | 7y 11m | M | Pug | V1 | 40d | 2 | 1.1795 | 82.45 |
| **170** | 4y 11m | M | Beagle | V3 | 19d | 0.5 | 0.387 | 36.8 |
| **171** | 6y 5m | F | Labrador Retriever | V2 | 7mo 6d | 1 | 0.614 | 42.92 |
| **172** | 1y 2m | F | Labrador Retriever | V3 | 1m 16d | 0.5 | 0.673 | 45.5 |
| **173** | 3y 9m | F | Labrador Retriever | V2 | 3m 03 d | 1 | 0.614 | 42.92 |
| **174** | 5y 11m | M | Cava-lon | V3 | 30d | 1 | 0.939 | 66.83 |
| **175** | 4m | M | Saint Bernard | V2 | 12d | <0.5 | 0.431 | 34.5 |
| **177** | 6y | F | Tibetan Terrier | V5 | 3m 10d | 4 | 0.8575 | 57.99 |
| **180** | 5m | M | Saint Bernard | V2 | 32d | <0.5 | 0.422 | 26 |
| **181** | 4y 2m | M(N) | Beagle | V5 | 4m 1d | 2 | 0.5575 | 37.7 |
| **182** | 5 y | M(N) | Shih Tzu | V5 | 94d | 2 | 1.3475 | 82.21 |
| **184** | 5y 3m | F | Labrador Retriever | V5 | 29d | 0.5 | 0.8115 | 59.32 |
| **185** | 8m | M | Doberman | V5 | 30d | 0.5 | 0.679 | 40.46 |
| **186** | 8m | F | Doberman | V2 | 30d | 0.5 | 0.5325 | 37.65 |
| **187** | 1y 11m | F | Labrador Retriever | V8 | 3m 3d | 4 | 0.6085 | 42 |
| **189** | 1y | M | Pekingese | V8 | 1m 19d | 4 | 0.479 | 45.33 |
| **190** | 1y 3m | M | Pug | V7 | 19d | 0.5 | 0.9965 | 72.84 |
| **192** | 1y 1 m | F | Pug | V6 | 5m 15d | 0.5 | 0.5215 | 39.27 |
| **193** | 1y 1m | F | Labrador Retriever | V3 | 6m 17d | 1 | 0.768 | 56.14 |
| **194** | 3y 3m | F | Chihuahua | V6 | 30d | 1 | 0.5745 | 45.05 |
| **195** | 1y | M | Siberian Husky | V5 | 3m 18d | 0.5 | 0.512 | 37.42 |
| **197** | 2y | M | Pug | V1 | 6m 24 | <0.5 | 0.6685 | 48.86 |
| **198** | 3y 7 m | F(N) | Labrador Retriever | V6 | 3m | 0.5 | 0.568 | 41.52 |
| **199** | 11y | M | Havanese | V6 | 6d | 8 | 0.741 | 70.13 |
| **200** | 8y 8m | F | Havanese | V8 | 10m 23 d | 1 | 0.829 | 50.57 |
| **201** | 6y 2m | M | Spitz | V8 | 1m 16d | 2 | 1.12 | 82.2 |
| **202** | 5m | F | Himalayan Shepherd | V8 | 22d | 2 | 0.4105 | 30 |
| **203** | 11y | F | Cocker Spaniel | V5 | 5m 17d | 1 | 0.5855 | 39.6 |
| **205** | 7y | M | Spitz | V2 | 3m 1d | 1 | 0.5405 | 39 |
| **206** | 4y 8m | F | Spitz | V5 | 597d | <0.5 | 0.421 | 35.45 |
| **207** | 7y 8m | M | Spitz | V6 | 2 y 3 m | 0.5 | 0.592 | 43.27 |
| **209** | 4y 6m | M | ND | V2 | 24d | 4 | 0.591 | 38.3 |
| **211** | 2y 1m | M | Labrador Retriever | V8 | 1m 23d | 2 | 0.514 | 50.29 |
| **212** | 5y | M(N) | ND | V8 | 1m 15d | 4 | 0.425 | 28.7 |
| **216** | 5y11m | M | Labrador Retriever | V1 | 1m 1d | 2 | 0.7355 | 53.76 |
| **219** | 7y 3m | F | Labrador Retriever | V5 | 11m 7 d | 2 | 0.5115 | 37.88 |
| **220** | 10m | M | Doberman | V5 | 3m 7d | 0.5 | 0.4375 | 31.98 |
| **221** | 10m | F | Doberman | V2 | 4m 7d | 0.5 | 0.5235 | 37.94 |
| **222** | 2y 1m | M | American Cocker Spaniel | V2 | 18d | 1 | 0.5615 | 41.04 |
| **223** | 1y 4 m | M | Shih Tzu | V2 | 14d | 8 | 0.62 | 65.49 |
| **224** | 5y 6 m | F | LS | V1 | 2m 12d | 4 | 0.4725 | 74.63 |
| **229** | 2y 4m | M | Pug | V3 | 17d | 4 | 0.897 | 62.92 |
| **230** | 9y | M | Shih Tzu | V6 | 14 d | 8 | 1.441 | 100 |
| **232** | 1y | M | Shih Tzu | V2 | 91 d | 4 | 0.6275 | 50.3 |
| **233** | 2y 8m | M(N) | LabraPor Retriever | V2 | 125 d | 4 | 0.942 | 57.47 |
| **234** | 9m | M | Dachshund | V6 | 213 d | 4 | 0.741 | 46.85 |
| **235** | 3y 2m | M | Golden Retriever | V6 | 146 d | 4 | 1.135 | 81.39 |
| **236** | 5y | M(N) | Labrador Retriever | V2 | 322 d | 1 | 0.555 | 37.53 |
| **237** | 5y 6 m | F | Labrador Retriever | V6 | 252 d | 4 | 0.879 | 37.53 |
| **239** | 1y 6m | F | Saint Bernard | V8 | 259 d | 1 | 0.6595 | 45.27 |
| **240** | 4y | M | Coton de Tullear | V3 | 339 d | 0.5 | 1.1885 | 80.38 |
| **242** | 5y 10m | F(N) | Golden Retriever | V2 | 33 d | 2 | 0.8185 | 55.36 |
| **243** | 5y 5m | M | Pug | V2 | 71 d | 0.5 | 0.867 | 60.83 |
| **245** | 4y 9m | F | Golden Retriever | V2 | 232d | 0.5 | 0.531 | 38.94 |
| **247** | 6y 5m | F | Beagle | V2 | 88 d | 4 | 0.5675 | 39.81 |
| **249** | 3y 6 m | M(N) | Siberian Husky | V2 | 410d | 1 | 0.963 | 70.39 |
| **250** | 7y | F | Toy Poodle | V5 | 295 d | 4 | 1.0563 | 73.39 |
| **251** | 5y 3m | F | Labrador Retriever | V7 | 39d | 0.5 | 0.366 | 32.17 |
| **252** | 11y | M(N) | Shih Tzu | V3 | 32d | 2 | 0.5745 | 50.5 |
| **253** | 1y 7m | F(N) | ND | V3 | 47 d | 2 | 0.669 | 58.81 |
| **254** | 1y 2m | M(N) | ND | V3 | 47 d | 2 | 0.7265 | 63.86 |
| **255** | 1y 2m | F(N) | ND | V3 | 47 d | 4 | 0.868 | 76.3 |
| **256** | 1y 2m | M(N) | ND | V3 | 47 d | 1 | 0.5725 | 50.32 |
| **257** | 8m | F(N) | ND | V3 | 47 d | 8 | 1.117 | 98.19 |
| **258** | 1y 2m | M(N) | ND | V3 | 47 d | 1 | 0.753 | 66.19 |
| **259** | 7m | M(N) | ND | V3 | 47 d | 2 | 0.4705 | 41.36 |
| **260** | 7m | F(N) | ND | V1 | 47 d | 0.5 | 0.3975 | 27.71 |
| **261** | 3y 8m | F | Spitz mix | V2 | 43d | <0.5 | 0.411 | 34.54 |
| **263** | 4y 3m | F(N) | Golden Retriever | V2 | 66 d | 4 | 0.55 | 39.13 |
| **265** | 5y 7m | M | Siberian Husky | V4 | 57 days | 4 | 0.7905 | 48.23 |
| **266** | 8m | M | Boxer | V2 | 141 d | 2 | 0.32 | 22.44 |
| **267** | 3y | M | Golden Retriever | V2 | 52 d | 1 | 0.585 | 51.42 |
| **268** | 1y 9 m | M | Beagle | V8 | 74 d | 0.5 | 0.46 | 48.74 |
| **269** | 2y 2m | M | Golden Retriever | V6 | 85 d | 4 | 0.8295 | 72.92 |
| **270** | 4y 11m | F | Golden Retriever | V6 | 39 d | 2 | 0.5085 | 44.7 |
| **272** | 17y 4 m | M (N) | ND | V2 | 209d | 0.5 | 0.4455 | 42.36 |
| **273** | 6y 2m | M (N) | Collie cross | V8 | 26d | 2 | 0.5975 | 52.52 |
| **274** | 1y 10m | F (N) | Toy Poodle | V7 | 310d | 0.5 | 0.2195 | 20.87 |
| **276** | 4y 9m | M | Labrador Retriever | V2 | 45d | 4 | 0.6055 | 59.24 |
| **277** | 2y 2m | F | Beagle | V7 | 93d | 0.5 | 0.896 | 54.74 |
| **278** | 2y 11m | M | Cocker Spaniel | V2 | 144d | 0.5 | 0.4415 | 41.9 |
| **279** | 4y 6m | M | Labrador Retriever | V8 | 65d | 0.5 | 0.65 | 55.87 |
| **280** | 5y | F | Labrador Retriever | V1 | 42d | <0.5 | 0.285 | 33.23 |
| **281** | 5y 6m | M | Collie | V8 | 43d | 1 | 0.585 | 51.42 |
| **282** | 3y | F | Pug | V3 | 42d | 4 | 0.51 | 48.5 |
| **284** | 9y | M(N) | Labrador Retriever | V3 | 83d | 4 | 0.5415 | 52.98 |
| **285** | 5y | M | Labrador Retriever | V6 | 136d | 4 | 1.054 | 92.65 |
| **287** | 5y 7m | F | Labrador Retriever | V8 | 30d | 4 | 0.7595 | 74.31 |
| **288** | 2y 3 m | M | ND | V8 | 30d | 0.5 | 0.425 | 40.36 |
| **289** | 1y 8m | M | ND | V8 | 37d | 0.5 | 0.4125 | 39.22 |
| **290** | 1y 3m | M | Cocker Spaniel | V8 | 30d | 2 | 0.578 | 50.81 |
| **291** | 3y 4m | F | Spitz mix | V2 | 34d | 0.5 | 0.6895 | 60.61 |
| **292** | 4y 5m | M (N) | Maltese Terrier | V8 | 32d | 1 | 0.687 | 67.27 |
| **293** | 1y 11m | F | Pomeranian | V3 | 217d | 1 | 0.8785 | 85.95 |
| **294** | 7y | M | Pug | V3 | 105d | 0.5 | 0.5415 | 55.01 |
| **295** | 5y | M(N) | ND | V2 | 1145d | <0.5 | 0.421 | 25.68 |
| **296** | 2y 10 m | M | Maltese Terrier | V2 | 31d | 4 | 0.74458 | 72.84 |
| **297** | 1y 10 m | F | Maltese Terrier | V2 | 26d | 2 | 0.7185 | 70.3 |
| **298** | 9y | M | Shiba Inu | V2 | 59d | 2 | 0.349 | 34.14 |
| **299** | 3y | M | Maltese Terrier Terrier | V8 | 79d | 4 | 0.9025 | 88.3 |
| **300** | 11m | M(N) | ND | V3 | 132d | 0.5 | 0.356 | 36.64 |
| **302** | 8y 5m | M | Labrador Retriever | V8 | 81d | 4 | 0.929 | 90.9 |
| **303** | 11y | M | Pug | V2 | 136d | 2 | 0.7525 | 73.63 |
| **305** | 1y | F | Shih Tzu | V2 | 260d | 1 | 0.739 | 45.08 |
| **306** | 9y 2 m | M | Labrador Retriever | V2 | 347d | 1 | 0.578 | 39.33 |
| **307** | 7y | F | Labrador Retriever | V3 | 90d | 16 | 1.9075 | 116.38 |
| **310** | 4.5m | M | Siberian Husky | V3 | 32d | 0.5 | 0.228 | 22.3 |
| **311** | 5m 10d | M | Siberian Husky | V3 | 41d | 1.0 | 0.2635 | 25.78 |
| **312** | 1y | F | Siberian Husky | V2 | 30d | 2 | 0.48 | 46.96 |

**Note: Gender**: M- Male, F- Female, N- Neutered
