# Supplementary material for: Evaluation of the immune status of dogs vaccinated against rabies by an enzyme-linked immunosorbent assay using crude preparations of insect cells infected with a recombinant baculovirus encoding the rabies virus glycoprotein gene
Source: PLoS One. 2024 Dec 3;19(12):e0314516. doi: 10.1371/journal.pone.0314516 (PMC11614288; doi:10.1371/journal.pone.0314516)
Supplement: S1 Raw images — (PPTX) [file pone.0314516.s002.pptx]

## Slide 1
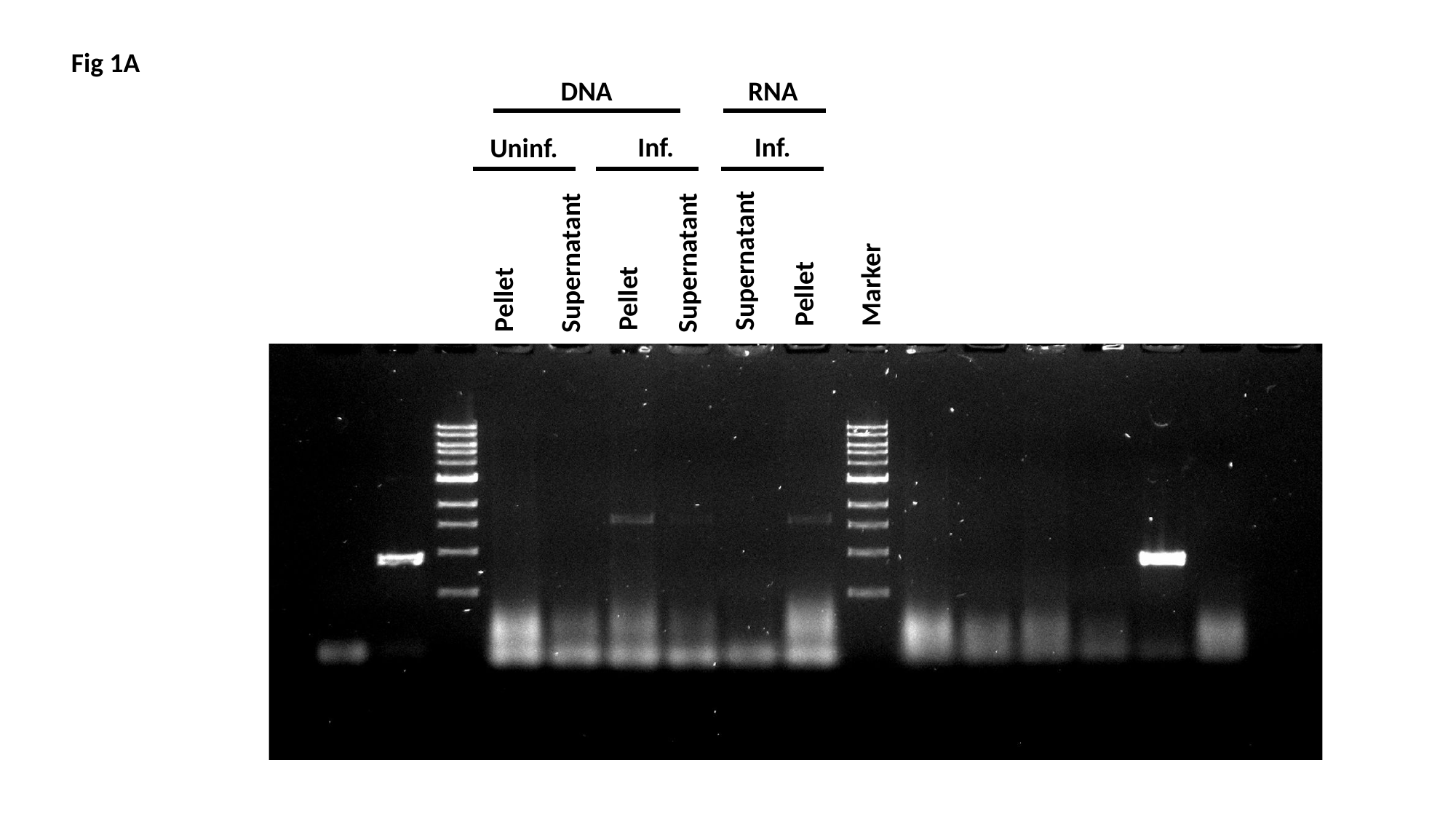

Fig 1A
RNA
DNA
Inf.
Inf.
Uninf.
Supernatant
Supernatant
Supernatant
Marker
Pellet
Pellet
Pellet

## Slide 2
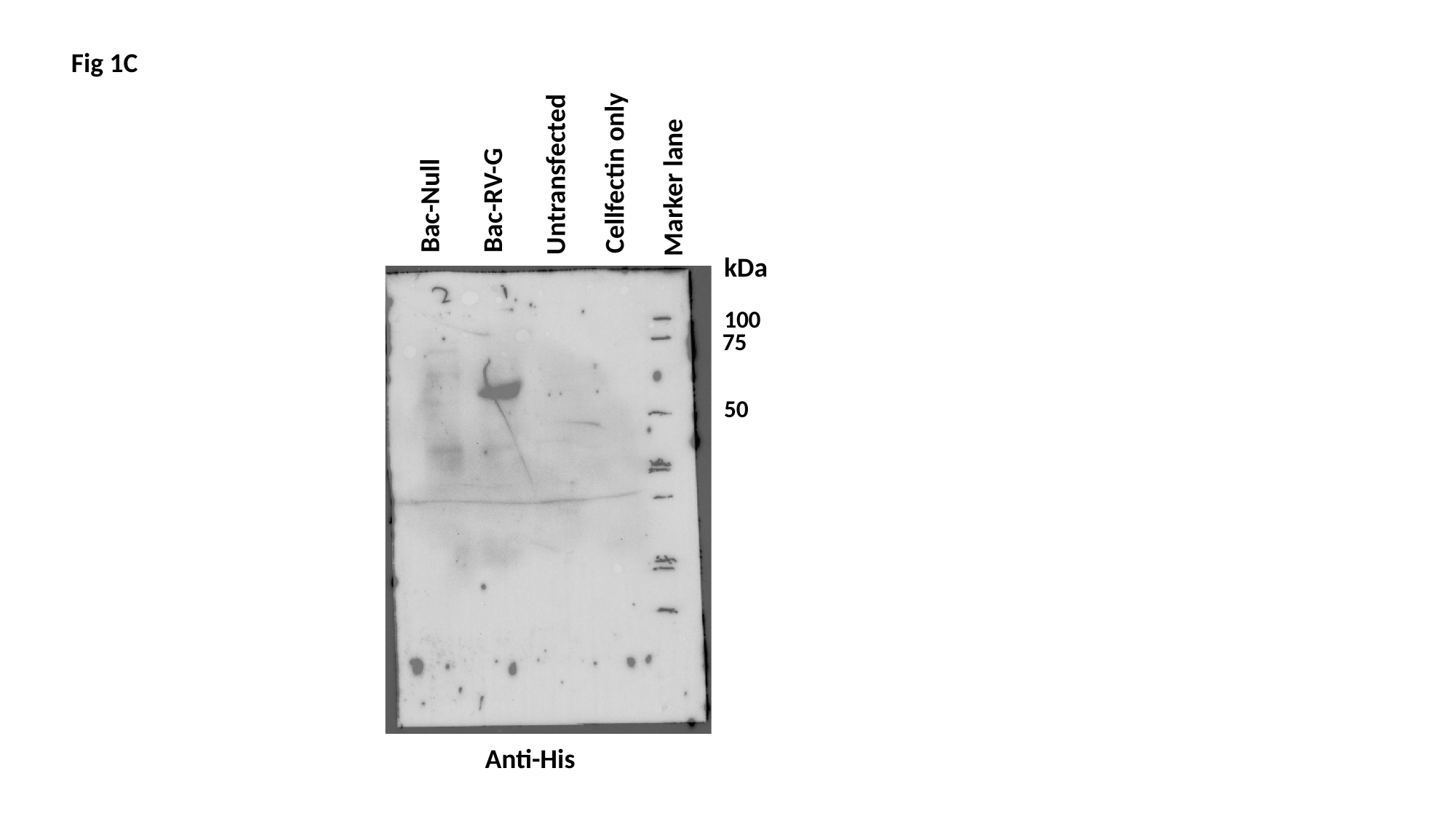

Fig 1C
Cellfectin only
Untransfected
Marker lane
Bac-RV-G
Bac-Null
kDa
100
75
50
 Anti-His

## Slide 3
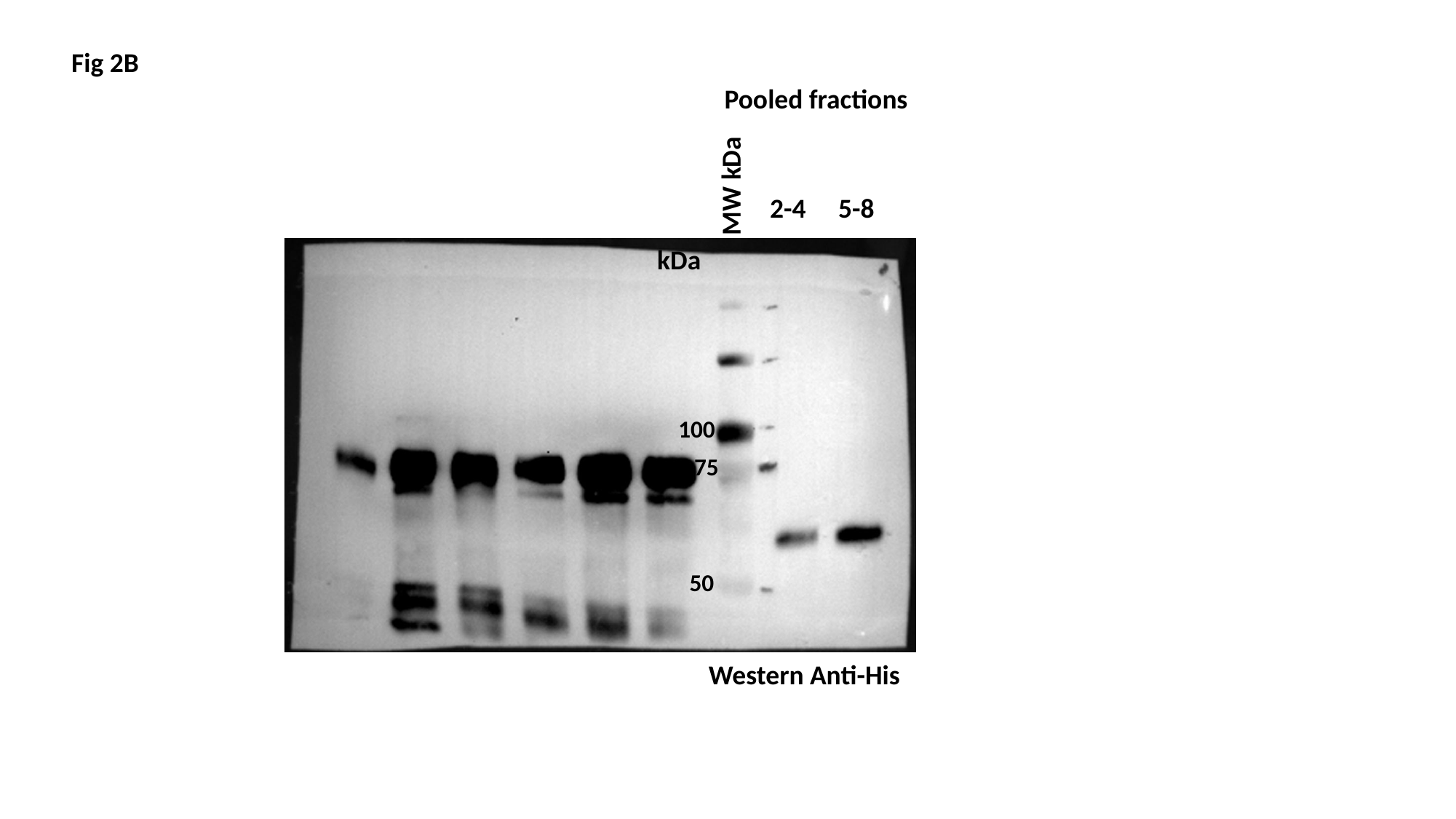

Fig 2B
Pooled fractions
MW kDa
5-8
2-4
kDa
100
75
50
Western Anti-His

## Slide 4
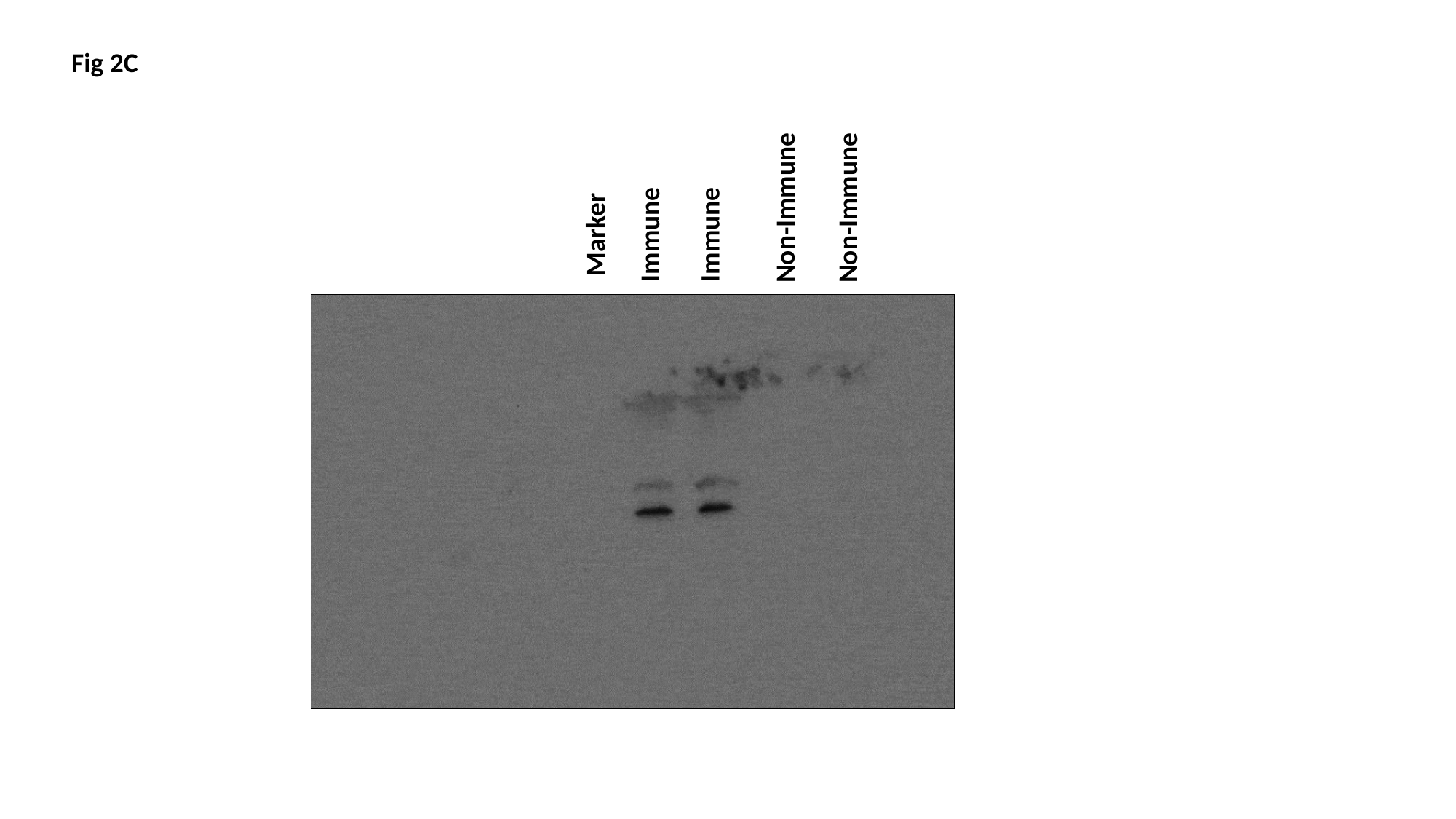

Fig 2C
Non-Immune
Non-Immune
Marker
Immune
Immune
