# Supplementary material for: Evaluation of the immune status of dogs vaccinated against rabies by an enzyme-linked immunosorbent assay using crude preparations of insect cells infected with a recombinant baculovirus encoding the rabies virus glycoprotein gene
Source: PLoS One. 2024 Dec 3;19(12):e0314516. doi: 10.1371/journal.pone.0314516 (PMC11614288; doi:10.1371/journal.pone.0314516)
Supplement: Attachment — Submitted filename: Responses to reviewers.docx [file pone.0314516.s003.docx]

**Responses to reviewers**

We thank the reviewer for a very detailed scrutiny of the manuscript. The suggestions have significantly improved the manuscript. Provided below are point by point responses to the suggestions.

**Reviewer #1**

1. Introduction: Include the new species name Lyssavirus rabies according to ICTV (<https://ictv.global/report/chapter/rhabdoviridae/rhabdoviridae/lyssavirus>). We have also changed the abbreviation of rabies virus from RV to RABV throughout the manuscript, in accordance with ICTV.

Response: This has been included right in the beginning of the Introduction (see line 47 in the clean copy of the revised manuscript).

2. Sentence Improvement: Improve the sentence “However, there is no means to verify the success of the vaccination programmes other than keeping a record of the vaccination.” to provide a clearer context.

Response: Context has been provided (see lines 55-58 in the clean copy of the revised manuscript)).

3. Cell Line Description: On page 10, line 206, specify which cell line was used and provide a detailed description.

Response: Cell line specification and description are now provided (see lines 247-250 in the clean copy of the revised manuscript)).

4. Figure 1: Ensure the order of the description of the wells matches the order shown in the image to avoid confusion for the readers.

Response: We thank the reviewer for pointing this out. The description has now been modified for all the three panels for clarity (see lines 298, 306 and 309 in the clean copy of the revised manuscript).

5. Figure 2C: Clarify which fraction was submitted by specifying “One of the fractions (indicate which) was submitted...”

Response: It was pooled fraction 5-8, and this has been specified now (see line 337 in the clean copy of the revised manuscript).

6. Table 3: Include a footnote indicating that the kappa (κ) value of 0.61–0.80 is considered “substantial agreement” and not “almost perfect agreement.”

Response: A foot note has been added to the Table (see lines 391-392 in the clean copy of the revised manuscript). It may be noted that we had stated it to be ‘good’ agreement in the description (see line 388 in the clean copy of the revised manuscript).

7. ELISA Antigen Explanation: Provide a better explanation as to why the purified G protein was not used to perform the ELISA, and describe the difficulties encountered in the purification step.

Response: A sentence is now included in the Discussion (see lines 419-423 in the clean copy of the revised manuscript).

8. Comparison with Other Studies: Include a comparison with values found in other studies:
   - Cliquet F, McElhinney LM, Servat A, Boucher JM, Lowings JP, Goddard T, Mansfield KL, Fooks AR (2004) Development of a qualitative indirect ELISA for the measurement of rabies virus-specific antibodies from vaccinated dogs and cats. J Virol Methods 117:1–8. https://doi.org/10.1016/j.jviromet.2003.12.001.
   - Cliquet F, Müller T, Mutinelli F, Geronutti S, Brochier B, Selhorst T, Schereffer JL, Krafft N, Burow J, Schameitat A, Schlüter H, Aubert M (2003) Standardisation and establishment of a rabies ELISA test in European laboratories for assessing the efficacy of oral fox vaccination campaigns. Vaccine 21:2986–2993. https://doi.org/10.1016/S0264-410X(03)00102-6.
   - Cliquet F, Sagné L, Schereffer JL, Aubert MF (2000) ELISA test for rabies antibody titration in orally vaccinated foxes sampled in the fields. Vaccine 18:3272–3279. https://doi.org/10.1016/S0264-410X(00)00127-4.
   - Wasniewski M, Cliquet F. Evaluation of ELISA for detection of rabies antibodies in domestic carnivores. J Virol Methods. 2012 Jan;179(1):166-75. doi: 10.1016/j.jviromet.2011.10.019.
   - Zajac MD (2019) Development and evaluation of a rabies enzyme-linked immunosorbent assay (ELISA) targeting IgM and IgG in human sera. https://doi.org/10.13140/RG.2.2.32365.54242.
   - Zhao R, Yu P, Shan Y, Thirumeni N, Li M, Lv Y, Li J, Ren W, Huang L, Wei J, Sun Y, Zhu W, Sun L (2019) Rabies virus glycoprotein serology ELISA for measurement of neutralizing antibodies in sera of vaccinated human subjects. Vaccine 37:6060–6067. <https://doi.org/10.1016/j.vaccine.2019.08.043>.

Response: We appreciate the reviewer for suggesting and drawing our attention to this. We have now added an entire paragraph in the Discussion on this (lines 471-503 in the clean copy of the revised manuscript), but have focussed on those studies which have tested for sera from animals, particularly carnivores. Although we agree with the importance of application of such tests in humans, in the context of our assay, studies using human sera only have been omitted.

9. Limitations of the Study: The authors should include a sentence in the discussion topic that reports the limitations of the study.

Response: Two sentences have been added at the end of Discussion (lines 508-511 in the clean copy of the revised manuscript).

10. In the study by Kramps et al. (1994), the authors demonstrated that their blocking ELISA was superior to a commercially available indirect ELISA and to the 24-hour virus neutralization test in detecting low antibody levels in serum. Additionally, this blocking ELISA was capable of detecting specific antibodies in serum as early as 7 days post-infection. Thus, it would be beneficial to evaluate whether the newly developed ELISA here can detect antibodies as early as 7 days post-infection, similar to Kramps et al. studies.
    Kramps JA, Magdalena J, Quak J, Weerdmeester K, Kaashoek MJ, Maris-Veldhuis MA, Rijsewijk FA, Keil G, van Oirschot JT. A simple, specific, and highly sensitive blocking enzyme-linked immunosorbent assay for detection of antibodies to bovine herpesvirus 1. J Clin Microbiol. 1994 Sep;32(9):2175-81. doi: 10.1128/jcm.32.9.2175-2181.1994.
    Kramps JA, Banks M, Beer M, Kerkhofs P, Perrin M, Wellenberg GJ, Oirschot JT. Evaluation of tests for antibodies against bovine herpesvirus 1 performed in national reference laboratories in Europe. Vet Microbiol. 2004 Sep 8;102(3-4):169-81. doi: 10.1016/j.vetmic.2004.07.003.

Response: Although we understand the argument for detection of antibodies as early as possible, we are not clear about the context of the studies suggested by the reviewer. They refer to bovine herpesvirus 1, and not to rabies. In addition, the context of the ELISA that we have developed is for monitoring antibodies after vaccination, whereas the mentioned studies about BHV-1 are after infection, which is impractical or inconsequential for rabies. The fundamental difference in these two cases is that the one for BHV-1 is for diagnostic purposes and the one for rabies is for monitoring purposes. Furthermore, early detection in case of rabies may not have a bearing on the potential to protect since early responses are dominated by IgM. It is thought that IgM is not as efficient as IgG against rabies virus due to its inability to penetrate interstitial tissue that has been impregnated with high levels of virus through the saliva in the bite of a rabid animal (Kennedy et al. 2007; Fooks 2018). Indeed, driving sustained IgG production is one of the main targets of vaccination against rabies (Flamand et al. 1993; Dorfmeier et al. 2013). Although we agree that a time-lapse experiment on the appearance of potentially protective antibodies is required, we feel that this discussion would be deviant from the focus of the manuscript.

References:

1. Dorfmeier, C.L.; Shen, S.; Tzvetkov, E.P.; McGettigan, J.P. 2013. Reinvestigating the role of IgM in rabies virus postexposure vaccination. J. Virol. 87:9217-9222.
2. Flamand, A.; Raux, H.; Gaudin, Y.; Ruigrok, R.W. 1993. Mechanisms of rabies virus neutralization. Virology 194:302-313.
3. Fooks, A. 2018. Rabies (Infection with RABV and Other Lyssaviruses). In Manual of Diagnotics Tests and Vaccines for Terrestrial Animals; World Organisation for Animal Health (OIE), Paris, France, 2018; pp. 578–612.
4. Kennedy, L.J.; Lunt, M.; Barnes, A.; McElhinney, L.; Fooks, A.R.; Baxter, D.N.; Ollier, W.E. 2007. Factors influencing the antibody response of dogs vaccinated against rabies. Vaccine 25:8500-8507.
